# Tumours of Nasal Septum: A Retrospective Study of 32 Patients

**DOI:** 10.3390/ijerph19031713

**Published:** 2022-02-02

**Authors:** Federico Sireci, Francesco Dispenza, Francesco Lorusso, Angelo Immordino, Palmira Immordino, Salvatore Gallina, Giorgio Peretti, Frank Rikki Canevari

**Affiliations:** 1 Otorhinolaryngology Section, Department of Biomedicine, Neuroscience and Advanced Diagnostics (BIND) University of Palermo, 90100 Palermo, Italy; francesco.dispenza@gmail.com (F.D.); dott.francescolorusso@gmail.com (F.L.); angelo.immordino182@gmail.com (A.I.); salvatore.gallina@unipa.it (S.G.); 2 Hygiene and Preventive Medicine Section, Department of Health Promotion, Maternal and Infant Care, Internal Medicine and Medical Specialities (PROMISE), University of Palermo, 90100 Palermo, Italy; palmira.immordino@unipa.it; 3 IRCCS Ospedale Policlinico San Martino, 16100 Genoa, Italy; giorgio.peretti@unige.it (G.P.); canevari@edu.unige.it (F.R.C.); 4 Unit of Otorhinolaryngology—Head and Neck Surgery, University of Genoa, 16100 Genoa, Italy

**Keywords:** nasal septum, benign tumour, malignant tumour

## Abstract

Objective: Tumours of the nasal septum are a rare and heterogeneous group of lesions in the sinonasal tract. The management of the different lesions of this site is debated. The aim of this study is to share our experience on a rare clinical condition and stimulate other centres to publish theirs. Methods: We retrospectively analysed the databases of sinonasal tumours treated at the Sections of Otolaryngology (ENT) of two University Hospitals (Palermo and Genova) between 2012 and 2020. Results: From the two databases, a cohort of 32 patients with tumours of nasal septum were selected. All patients underwent an endoscopic examination. Large tumours underwent preoperative computed tomography (CT) scan without contrast medium. In 22 (68.7%) cases, the preoperative radiologic evaluation also included magnetic resonance imaging (MRI) with gadolinium to obtain a better differentiation of the lesions and study the vascular pattern. All the large lesions were biopsied under endoscopic guidance using local anaesthesia; the same approach was used to remove the tumours and their attachment with safe resection margins. Conclusions: While malignant lesions require an excision of the mass with resection of all layers of the nasal septum, benign lesions must be typed according to histological considerations in order to plan the most appropriate type of surgical resection.

## 1. Introduction

Tumours of the nasal septum belong to a rare and heterogeneous group of lesions in the sinonasal tract. According to the published literature, the incidence of benign neoplasms is not known since many of them occur sporadically in the sinonasal area [1]. Lobular capillary haemangioma (pyogenic granuloma) seems to be the most frequent, with a percentage between 7 and 29% [2], followed by inverted papilloma (2.5%) and pleomorphic adenoma [3]. Scarce information is available about malignancies, representing 9% of all sinonasal cancers [4] and being more common in males and persons older than 50 years. The incidence and histological type can vary in different geographical areas, probably due to occupational, social and genetic factors. As a result, the distribution in the literature is variable [5]. The management of the different lesions of this site is debated because the resection of all the layers of nasal septum (mucoperiosteum/mucoperichondrium bilateral with the cartilage/bone) or some of these (mucoperiosteum/mucoperichondrium unilateral with or without the cartilage/bone) is not coded by the guidelines [6,7]. For this reason, the aim of this study is to present our experience about the tumours of nasal septum and try to standardize the management and surgical approach to these lesions.

## 2. Materials and Method

### 2.1. Study Design

We performed a retrospective analysis of the databases of sinonasal tumours treated at the Sections of Otolaryngology of two University Hospitals (Palermo and Genova) between 2012 and 2020. Only primary tumours with origin from the nasal septum were included in the study. Clinical, demographic, histologic, radiographic characteristics, as well as operative and post-operative findings, complications, adjuvant therapy and follow-up data were analysed.

### 2.2. Pre-Operative Workup and Surgical Treatment

All patients underwent endonasal endoscopy to evaluate the extension of the neoplasm. Small lesions (i.e., diameter ≤ 2 mm) were removed by endoscopic resection in local anaesthesia without imaging. In the large tumours (i.e., diameter > 2 mm), a CT scan and/or a contrast-enhanced MRI were/was performed to study the lesions, if imaging was not clear about the involvement of other anatomical structures and/or endoscopy did not show a secure implant base, a biopsy under local anaesthesia was always performed, to be followed by endoscopic resection in general anaesthesia. About the malignancies, the adjuvant therapy was discussed with multidisciplinary team, and it was used for the cases with involved margins, advanced staging (III–IV) or grading (high grade) and involvement of critical regions, such as frontal sinus, sphenoid sinus, anterior skull base (with or without dural infiltration), lamina papyracea or periorbit and nasopharynx.

### 2.3. Follow-Up

Postoperative follow-up was performed with nasal endoscopy every 15 days until complete healing of the surgical cavity, every 3 months during the first year and every 6 months thereafter. Post-operative MRI with gadolinium was performed 6 months after surgery and every year thereafter for the large tumours. For the malignancy, systemic staging through total body positron emission tomography (PET) or total body CT scan and ultrasound of the neck was performed once a year. In the small lesions, an endoscopic exam was considered to be enough.

## 3. Results

A cohort of 32 patients with tumours of the nasal septum were detected, including 19 males and 13 females (M:F = 1.5:1). The mean age was about 56.24 years (SD ± 14.69 years) with a range from 14 to 86 years.

In 17 patients (53.1%), the symptom was unilateral nasal obstruction, 10 (31.2%) reported unilateral epistaxis and 4 (12.5%) reported unilateral epistaxis and nasal obstruction [8]. One (3.1%) patient was asymptomatic. In 19 patients (59.4%), the lesion was on the right nostril, in 12 (37.5%) on the left and in 1 (3.1%) case bilateral.

All patients with large tumours underwent a preoperative CT scan without contrast medium to study the bone anatomical limits that can be invaded by the lesion. Often the contrast medium can give information on vascularization, but it is not useful for the typization of the tumour. In 22 (68.7%) cases, the preoperative radiologic evaluation also included an MRI with gadolinium to obtain a better differentiation of the lesion and to study the vascular pattern. In cases of malignancies, the presence of local or distant metastases was assessed with a total-body contrast-enhanced CT scan and ultrasound of the neck.

In cases of small tumours, an endoscopic resection was performed, while in the large lesions, a biopsy preceded the surgery.

All the specimens were sent to the Department of Human Pathology for histopathological diagnosis. A total of 28 (87.5%) cases were benign neoplasm, of which 9 (28.1%) patients had a lobular capillary haemangioma (LCH), also called pyogenic granuloma; 13 (40.6%) had an inverted papilloma (IP); and 1 (3.1%) case of Schwannoma, 1 Warthin’s tumour (WT), 1 respiratory epithelial adenomatoid hamartoma (REAH), 1 Pleomorphic Adenoma (PA), 1 blue nevus (BN) and 1 solitary fibrous tumour (SFT). Four (12.5%) cases were malignant tumours, of which one was Mucosal Melanoma (MM), one was non-Intestinal-type adenocarcinoma (n-ITAC), one was poorly differentiated non-keratinizing squamous cell carcinoma (SCC) and one was low grade chondrosarcoma (Table 1). Based on the clinical, radiological and histopathological findings, all neoplasms were staged according to the system of Krouse for inverted papilloma [9] and the 2016 Union for International Cancer Control TNM classification [10] for the malignancies.

The biopsied cases with histological diagnosis of benign neoplasm underwent endoscopic resection, whereas, in cases of malignancies, the presence of local or distant metastases was assessed with a total-body contrast-enhanced CT scan and an ultrasound of the neck. Treatment planning based on a common management strategy was discussed by the multidisciplinary team. In the cases of benign tumours, a partial resection of the nasal septum was performed. In particular:

- In the lobular capillary haemangioma, only the mucoperichondrium was removed.
- In the others lesions, the mucoperichondrium with cartilage and/or bone was removed.

In all malignant tumours, an endoscopic endonasal resection without external approach was possible, and a resection of all layers of nasal septum was performed [8]. In some of these cases, a resection of the nasal septum was not enough, and therefore the resection was completed by other surgical steps: nasopharyngeal endoscopic resection (NER) type II for the n-ITAC; bilateral medial maxillectomy for the chondrosarcoma; and bilateral selective neck dissection (SND I-III) for the MM. No intraoperative or postoperative complications occurred. In all patients, nasal packing was removed within 48 h, and the antibiotic therapy (i.e., intravenous cephalosporin) was administered the day before surgery and continued for at least 5 days. Irrigations with saline solution (twice a day) were recommended for at least 1 month [11]. Two malignant cases (mucosal melanoma and n-ITAC) were subjected to postoperative adjuvant radiotherapy (RT). Follow-up data were available for all patients and ranged from 12 to 60 (mean, 28.2) months. From the follow-up carried out, all patients have been disease free to date.

## 4. Discussion

**Figure 1:**
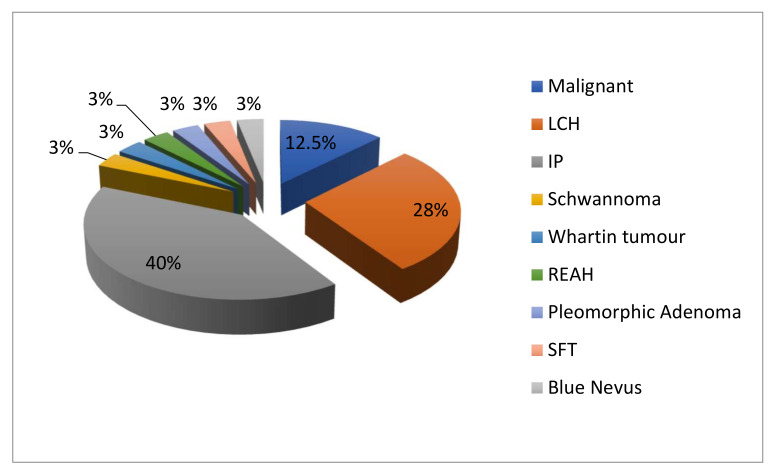
Pie chart that highlights the percentages of the different tumours of our series. LCH: Lobular capillary haemangioma; IP: Inverted Papilloma; SFT: solitary fibrous tumour; REAH: Sinonasal respiratory epithelial adenomatoid hamartoma.

The nasal septum is supported by a plate of hyaline cartilage (quadrangular cartilage) and a plate of bone (perpendicular lamina of ethmoid and vomer) covered by respiratory epithelium (pseudostratified ciliated columnar). This structure is mainly made ofciliated columnar and goblet cells attached to a basal lamina. Basal cells are observed just above the basal lamina. Septal glands with their ducts, vessels and nerves are present in lamina propria or rather mucoperiosteum/mucoperichondrium. The glands are not the same in all the septums: the anterior glands of the nasal septum are tubulo-alveolar with a serous secretion; the posterior glands are of the branched acinar type with a mucoid secretion. They lie partly within the vomeronasal capsule and most of the ducts of the posterior glands open into the cavity of the vomeronasal organ along the groove between the olfactory and columnar epithelia [12]. To synthesise our experience and the fragmented literature on the tumours of nasal septum, we started from histological considerations. In our cohort, 87.5% of the cases were benign tumours vs. 12.5% of malignant lesions, in line with existing published literature (Figure 1; Table 2).

The most frequent tumour was lobular capillary haemangioma (LCH) (28.1%; M:F = 2:7). This one, also known as pyogenic granuloma, is a benign vascular tumour with a microscopically distinctive lobular architecture that affects the skin and mucous membranes of the oral cavity and nasal region. The disease may appear in all ages. According to published literature, the incidence of this lesion is variable (7–29%), probably because its presence may be underestimated as it is confused with a bleeding angioma, and therefore small LCHs are simply electrocauterized. Usually, this lesion, if smaller than 1 cm, arises from the anterior portion of the nasal septum. In this case, imaging studies are not indicated, unless a differential diagnosis is needed (e.g., angiofibroma, angiomatous polyp, haemangioma, hemangiopericytoma, paraganglioma, angiosarcoma and highly vascularised metastases such as kidney, thyroid, lung or breast; schwannoma with Antoni A histological pattern). Endoscopic surgery is the treatment of choice for LCH. In particular, small size lesions, as in our cases, can be resected easily under local anaesthesia. Since the lesion is benign and the vessels of the nasal septum are contained in the mucopericondrium/periosteal, Puxeddu et al. suggest a radical resection on the subperichondrial or a subperiosteal plane with a margin of normal mucosa all around [13].

About benign epithelial lesions, the IP was present in 40.6% of cases, followed by 2 cases of salivary-gland-type adenomas (one PA and one WT) and 1 case of REAH. This high incidence compared to the available literature analysed (40% vs. 2.5%) is explained by the fact that our centres represent the local referral hospital for neoplastic pathology of the nasosinusal district, and therefore, we receive the most complex cases (e.g., IP) for surgical treatment. Usually, IP arises primarily from the lateral nasal wall and rarely from the nasal septum. Likely, focal hyperostosis, osteitis or bone remodelling are CT radiographic predictors of tumour origin or attachment. Histologically, IP shows an epithelium inverting into the stroma but with a distinct and intact basement membrane that separates both. However, the tumour may be associated with atypia, dysplasia and carcinoma in situ, as well as frank squamous cell carcinoma identified focally only with the definitive histological examination also in the area of hyperostosis. For this reason, endoscopic resection must foresee the excision of mucoperhicondrium/ostium with cartilage/bone with safety margin [14].

Salivary gland-type adenomas arise from minor salivary glands of the sinonasal tract. In particular, PA must be carefully assessed because a malignant transformation into carcinoma ex-pleomorophic adenoma (CXPA) occurs in approximately 6% of cases. Considering the presence of the glands in the stroma, the literature suggests a complete surgical excision of the tumour with clear resection margins by endonasal endoscopic resection of the mucoperhicondrium/ostium. Luckily, the treated PA of the nasal septum are significantly associated with a low prevalence of recurrence compared to tumour originating from the paranasal sinus [15,16]. Our case of WT of nasal septum is the first in the literature. It seems to originate from the undifferentiated epithelium of the intercalated ducts of the glands. The pathogenesis of these tumours is uncertain. Clinically, they may be similar to a mucocele. Compared to PA, these lesions are mainly benign without a possible evolution into malignant lesions. Considering that they belong to the same histopathological category of PA, the approach suggested is the same [17].

Another benign epithelial tumour is the respiratory epithelial adenomatoid hamartoma (REAH). Hamartoma can grow out in each part of the body and can be defined as epithelial, mesenchymal and mixed. In the sinonasal region, the majority of them are purely of epithelial type and are called REAHs. Its origin is from the surface epithelium with glandular elements arising from this epithelium and not from seromucous glands (unlike salivary-gland-type adenomas). They may appear as either isolated lesions or in association with inflammatory processes such as sinonasal polyposis. The majority of REAHs arise from the posterior nasal septum like our case, but some cases may arise from the olfactory cleft, middle meatus, inferior turbinate, maxillary sinus, nasopharynx and sphenoid sinus. Our case of REAH was in the right nostril between the septum and the olfactory cleft [15]. Differently from IP, the benign epithelial origin of this lesion suggests a muchoperichondral excision. In fact, both surgical treatments (less and more aggressive) resulted indeed to be effective to remove the REAH, and therefore a less aggressive and more conservative approach is sufficient for dealing with this kind of lesions [18,19].

The only “Benign soft tissue tumours” present in our records was the Schwannoma [20].

It is a neurogenic, slowly growing tumour which originates from Schwann cells of the sheath of the peripheral nerves. It can arise from any peripheral cranial nerve, with the exception of the olfactory and optic nerves. Only 4% of the head and neck Schwannomas originate from the nasal cavity and paranasal sinuses, often arising from the ophthalmic-maxillary branches of the trigeminal nerve and from the sympathetic parasympathetic nerves [19]. The risk of malignancy for Schwannomas is very low and increases predominantly to 10–15% in Von Recklinghausen’s disease [21].

According to the literature, as well as in our cases, the most frequent site of Schwannoma is the posterior nasal septum followed by the midportion and anterior nasal septum. Although it is unusual to identify a particular nerve of origin, the possible source for nasal septal schwannoma includes the sympathetic nerve to the septal blood vessels, the parasympathetic nerve to the septal mucous glands and the sensory nerve to the septum. Actually, there are no studies explaining the reason why nasal septal schwannomas arise from the posterior portion of the nasal septum, but considering that nerves are present in the stroma, an excision of the mucoperhicondrium/ostium extended to cartilage/bone is mandatory to be effective in this case.

**Figure 2:**
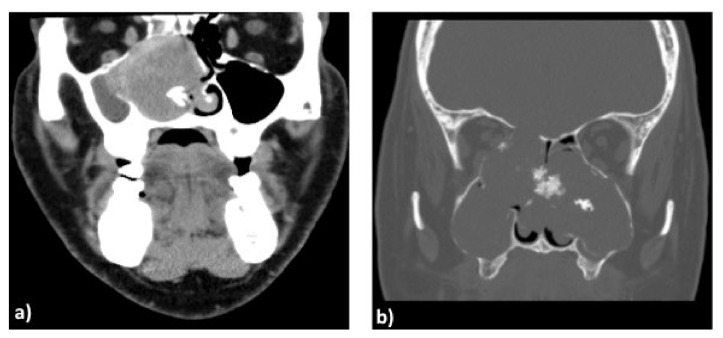
Coronal computed tomography scan showing a large solitary fibrous tumour (SFT) of the right nostril (**a**) and bilateral chondrosarcoma (**b**). The hyperostosis of nasal septum indicates the origin of SFT that compresses the medial wall of maxillary sinus, while in (**b**) it is showed the invasion of maxillary sinus bilaterally.

Concerning the “Borderline and low malignant potential tumours of soft tissue”, the only case was an extrapleural solitary fibrous tumour (SFT). This lesion typically involves the pleura but can rarely be present in another district. It is defined as a fibroblastic/myofibroblastic neoplasm, slowly growing, rarely metastasizing and with intermediate biological behaviour. Although most SFTs have a good prognosis, about 10–40% of cases relapse or metastasize. For instance, its clinically aggressive behaviour is related to the pathological criteria of malignancy established by the World Health Organization (WHO) as follows: presence of hypercellularity, increased mitoses (>4 mitoses per 10 high power elds), cytological atypia, tumour necrosis and/or infiltrative margins. In our case, the patient had benign histological criteria, and therefore, surgery without chemoradiotherapy was the treatment of choice. In particular, an endoscopic resection of the mass with excision of its attachment to the mucoperichondrium with cartilage was performed because, also in this lesion, malignancy is established only in the definitive histological examination, and the cartilage/bone often present hyperostosis [22] (Figure 2a).

Out of the 32 cases, 2 of them were “neuroectodermal tumours”: a blue nevus (BN, benign) and a mucosal melanoma (MM, malignant). The BN is a very rare lesion (only 5 cases are described in the literature) and goes in the differential diagnosis of pigmented lesions. It is characteristically small and asymptomatic, and the diagnosis is only histological. Treatment is complete excision (bilateral mucopericondrium and cartilage) for diagnostic and therapeutic purposes. The risk of recurrence is very low. Differently, in case of MM, patients often come late to medical observation because it grows asymptomatically until the last disease course. This lesion is rare (accounting for between 0.3% and 2% of all malignant melanomas and about 4% of head and neck melanomas) and more frequent in black patients. The primary approach is surgical resection [23]. Postoperative radiotherapy may help local control but does not affect survival. Prophylactic neck dissection in an N0 neck is not recommended as the incidence of node metastases is relatively low. Our patient presented cN2c in the imaging, and therefore, a bilateral selective neck dissection (SND I-III) was performed. One of the challenges of melanoma is the local recurrence. A long-term follow-up is indeed mandatory. When it occurs in sinonasal district, it is associated with poor survival rates, except for locations on the nasal septum where prognosis is better. In line with international literature [24], our case of MM was treated with an endoscopic resection of the mass with all layers of the nasal septum. Although histopathological examination evidenced free margins, due to the presence of pN2c, adjuvant radiotherapy was performed to improve local control of the primary lesion. This case is to date free of disease.

Concerning the “Malignant tumours of bone and cartilage”, the only case present was a low-grade chondrosarcoma. This lesion is a slowly growing malignancy of cartilage that arises from remnants of cartilage after ossification [25]. Three different gradings are possible, and this parameter with resection margins is the most important predictors of local recurrence and metastasis.

If possible, en-bloc excision is the preferred surgical treatment because these lesions are partially radio-resistant. Chondrosarcomas, especially low-grade tumours, are associated with an excellent prognosis if the lesions are completely resected. Our case showed an involvement of bilateral layers of nasal septum and bilateral invasion of the medial wall of the maxillary sinus (Figure 2b). Therefore, according to the literature, when these lesions involve the septum and the posterior septum and sphenoid rostrum without skull base or orbital involvement, they can be treated with a total endoscopic resection, including all layers of the nasal septum. Since the tumour histologically origins from cartilage, contralateral muchopericondrium must be removed to have margins free. Adjuvant radiotherapy was performed because the histopathology evidenced free margins and low grading [26,27,28,29]. Concerning the “malignant epithelial tumours”, we had two cases: a patient with a non-intestinal-type adenocarcinoma (n-ITAC) and Squamous Cell Carcinoma. While n-ITACs with ITAC are a glandular malignancy with a different prognosis, ITAC is generally locally aggressive with a local recurrence rate of around 50%, local lymph node spread in about 10% of the cases, a distant metastasis rate of 20% [29,30,31,32] and the 5-year survival is 40 to 60%. N-ITACs are divided into low- and high-grade subtypes. Low-grade N-ITAC has a more indolent course with an excellent prognosis, with 5-year survival up to 85%, while high-grade tumours may present extension of the neighbouring districts and have a very poor prognosis, with a 3-year survival of around 20% [33]. In our case, the tumour was a low-grade n-ITAC arising from vomer and involving rhinopharynx with the erosion of basisphenoid bone. Therefore, an endoscopic resection of the mass with all layers of the nasal septum with margins free and a nasopharyngeal endoscopic resection type II (NER 2) was performed. Although the tumour was margins free and low grade, it involved a critical region (sphenoid sinus and nasopharynx), and therefore, adjuvant radiotherapy was performed.

Squamous cell carcinoma (SCC) is the most common malignancy of the sinonasal tract. The peak incidence is between 60 and 70 years of age and it is more frequent in men than women (M:F = 2:1) [34]. An association between SCC and nickel exposure has been shown by Pedersen et al. Inverted papilloma is also associated with SCC in approximately 10% of the cases and can be synchronous (7.1%) or metachronous (3.6%) [35,36]. Nodal metastasis in paranasal SCCs are associated with a poor prognosis. SCC of the paranasal sinuses has a high propensity for neural invasion. Our case was a primary SCC of the vomer. An endoscopic resection with all layers of the nasal septum was performed. Adjuvant radiotherapy was not performed because margins were free, and the stage was II without the involvement of critical regions or of the lymph nodes.

## 5. Conclusions

**Figure 3:**
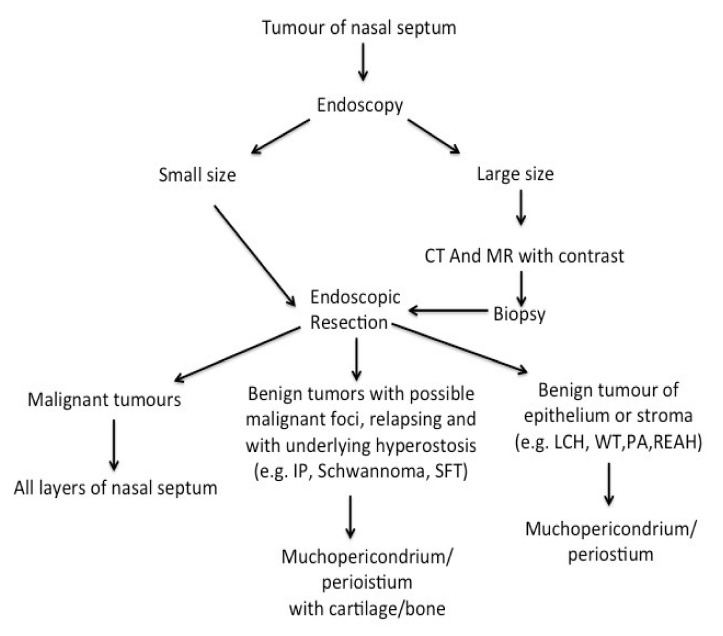
Flow diagram for management of tumour of nasal septum.

Nasal septal tumours are a heterogeneous group of rare lesions even compared to other neoplasms of the nasosinusal tract. CT and RM are the gold standard for diagnosis overall for large lesions in which a biopsy is mandatory. While malignant lesions require an excision of the mass with resection of all layers of the nasal septum, benign lesion must be typed according to histological considerations in order to plan the most appropriate type of surgical resection. In line with the available literature in the field, we performed a mucoperichondrium with cartilage/bone excision in patients with IP, Schwannoma and SFT, while an excision of mucoperichondrium was performed for LCH, WT, PA and REAH (Figure 3). The endoscopic approach is indicated for a minor comorbidity and post-operative hospitalization. In our experience, when margins were free, there was no recurrence of the disease in the follow-up. A multicentre study could allow us to identify more precise guidelines for the management of these neoplasms.

## Figures and Tables

**Table 1:** Details of patients who underwent endoscopic exeresis of tumour of nasal septum.

Patients (N°)	Gender/Age	Histology	Side (Left/Right/Bilateral)	Type of Septum Resection
1	F/72	LCH	Right	MP
2	M/44	Inverted Papilloma	Right	MPwC
3	M/56	n-ITAC	Left	NS
4	M/51	Inverted Papilloma	Right	MPwC
5	F/54	LCH	Left	MPwC
6	M/65	Inverted Papilloma	Right	MPwC
7	M/73	Schwannoma	Right	MPwC
8	M/58	Inverted Papilloma	Left	MPwB
9	F/67	Chondrosarcoma	Bilateral	NS
10	F/51	Inverted Papilloma	Right	MPwB
11	F/54	Warthin Tumour	Right	MP
12	M/71	Inverted Papilloma	Right	MPwC
13	F/86	LCH	Right	MP
14	M/69	Inverted Papilloma	Right	MPwC
15	F/75	Inverted Papilloma	Right	MPwB
16	F/54	Blue Nevus	Left	NS
17	M/46	Inverted Papilloma	Left	MPwC
18	M/57	SCC	Right	NS
19	M/67	Mucosal Melanoma	Right	NS
20	M/68	Inverted Papilloma	Left	MPwC
21	M/32	Inverted Papilloma	Left	MPwC
22	M/41	Inverted Papilloma	Left	MPwC
23	M/62	Inverted Papilloma	Left	MPwC
24	M/58	LCH	Right	MP
25	M/60	SFT	Right	MPwB
26	F/55	LCH	Right	MP
27	F/57	LCH	Left	MP
28	F/50	LCH	Right	MP
29	F/25	LCH	Left	MP
30	M/69	LCH	Right	MP
31	F/14	Pleomorphic Adenoma	Left	MP
32	M/46	REAH	Right	MP

F: Female; M: Male; LCH: Lobular capillary haemangioma; n-ITAC: non-intestinal-type adenocarcinoma; SCC: Squamous Cell Carcinoma; SFT: solitary fibrous tumour; REAH: Sinonasal respiratory epithelial adenomatoid hamartoma; MP: Mucoperichondrium; MPwC/B: Mucoperichondrium with Cartilage/Bone; NS: Nasal Septum.

**Table 2:** Synthesis of the main characteristics present in the literature of the tumours found in our series [1].

WHO Classifications of Tumours	Histopathology	Most Frequent Origin in the Nose	Local Recurrence	Malignant Trasformation	Therapy	Septal Resection
Benign Vascular Tumours	LCH	Septum	/	No	Surgery	MP
Benign epithelial Tumours	Inverted papilloma	Lateral wall of nasal cavity	Yes (0–78%)	Yes (3.6%)	Surgery	MPwC/B
	Plemorphic Adenoma	Septum	/	Yes (6%)	Surgery	MP
	Whartin’s Tumours	/	/	No	Surgery	MP
	REAH	Posterior Nasal Septum	/	No	Surgery	MP
Benign soft tissue tumours	Schwannoma	Naso ethmoid compartment	/	Yes (10–15% in Von Recklinghausen’s disease)	Surgery	MPwC/B
Borderline and low malignant potential tumours of soft tissue	SFT	Nasal cavity	/	Yes (10–20%)	Surgery	MPwC/B
Neuro-ectodermal Tumours	Blue nevus	Nasal cavity	Low	No	Surgery	NS
	Mucosal melanoma	Nasal cavity	High	-	Surgery ± RT	NS
Malignant tumours of bone and cartilage	Low grade chondrosarcoma	Maxillary Sinus	/	-	Surgery ± RT	NS
Malignant epitelial tumours	n-ITAC	Ethmoid sinus	/	-	Surgery ± RT	NS
	Squamous cell carcionma	Ethmoid sinus	Yes (20%)	-	Surgery ± RT	NS

LCH: Lobular capillary haemangioma; n-ITAC: non-intestinal-type adenocarcinoma; SFT: solitary fibrous tumour; REAH: Sinonasal respiratory epithelial adenomatoid hamartoma; MP: Mucoperichondrium; MPwC/B: Mucoperichondrium with Cartilage/Bone; NS: Nasal Septum; RT: radiotherapy; “/”: missing data; “-”: feature already present.

## Data Availability

The data are not publicly available due to privacy restrictions.
